# Environmental determinants of spatial and temporal variations in the transmission of *Toxoplasma gondii* in its definitive hosts^☆^

**DOI:** 10.1016/j.ijppaw.2013.09.006

**Published:** 2013-09-23

**Authors:** Eve Afonso, Estelle Germain, Marie-Lazarine Poulle, Sandrine Ruette, Sébastien Devillard, Ludovic Say, Isabelle Villena, Dominique Aubert, Emmanuelle Gilot-Fromont

**Affiliations:** a Université de Lyon, Université Lyon 1, Laboratoire de Biométrie et Biologie Evolutive UMR 5558, 43 bd du 11 novembre 1918, 69622 Villeurbanne Cedex, France; b CROC, Carnivores Recherche Observation Communication, 57590 Fonteny, France; c Université de Reims Champagne-Ardenne, UFR Médecine, SFR CAP-Santé, EA3800, 51 rue Cognacq Jay, 51095 Reims Cedex, France; d Université de Reims Champagne-Ardenne, Centre de Recherche et de Formation en Eco-éthologie, 08240 Boult-aux-Bois, France; e Office National de la Chasse et de la Faune Sauvage, CNERA PAD, Montfort, 01330 Birieux, France; f Université de Lyon, VetAgro-sup, Département de Santé Publique Vétérinaire, 1 avenue Bourgelat, 69280 Marcy l’Etoile, France

**Keywords:** Toxoplasmosis, Meteorological variations, Farm density, North-Atlantic Oscillation index, *Felis silvestris*, *Felis catus*

## Abstract

Toxoplasmosis is a major zoonosis, and its prevention requires multiple approaches due to the complex life-cycle of its causative agent, *Toxoplasma gondii*. Environmental contamination by oocysts is a key factor in the transmission of *T. gondii* to both humans and meat-producing animals; however, its spatial and temporal variations are poorly understood. We analysed the distribution of *T. gondii* seropositivity in a sample of 210 cats, including the European wildcat (*Felis silvestris silvestris*), the domestic cat (*Felis silvestris catus*) and their hybrids that were collected in Central and Eastern France between 1996 and 2006. We searched for spatial variability among communes and temporal variations among years to relate this variability to landscape and meteorological conditions, which can affect the population dynamics of rodent hosts and the survival of oocysts. The overall seroprevalence was 65.2% (95% CI: 58.6–71.4). As expected, adults were more often infected than young individuals, while the occurrence of infection was not related to cat genotypes. Seroprevalence correlated significantly with farm density and the North-Atlantic Oscillation index, which describes temporal variations of meteorological conditions at the continental scale. The highest seroprevalence values were obtained in areas with high farm densities and during years with cool and moist winters. These results suggest that both farming areas and years with cool and wet winters are associated with increased *T. gondii* seroprevalence in cats. As cat infection determines the environmental contamination by oocysts, climate and landscape characteristics should be taken into account to improve the risk analysis and prevention of *T. gondii*.

## Highlights

• *Toxoplasma gondii* seroprevalences in 112 European wildcats, 20 domestic cats and 47 hybrids are reported.
• The seroprevalence (overall 65.2%) did not differ with the type of cat.
• High farm densities and mild winters are associated with the highest seroprevalence.

## Graphical abstract

**Figure:**
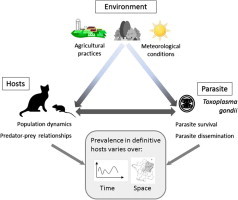

## 1 Introduction

Environmental factors such as landscape composition and climatic variations are known to influence the transmission of parasites with a complex life-cycle by acting on both the composition of animal communities and the predator–prey interactions between definitive and intermediate hosts (Patz et al., 2000; Daszak et al., 2001; Stenseth et al., 2002a). For instance, the dynamics of small mammal populations vary with the size and spatial array of their optimal habitat patches within a landscape (Lidicker, 1995; Giraudoux et al., 2003). Meteorological variations can also influence small mammal population dynamics by affecting primary productivity, leading to local declines in severe winters or high densities after warm and wet winters (Ottersen et al., 2001; Stenseth et al., 2002b). Such variations in the abundances of small mammals have been related to the dietary responses and population dynamics of several predators (Stenseth et al., 1999; Malo et al., 2004; Raoul et al., 2010). Some parasites with a complex life-cycle are characterised by a free-living stage that disseminates into the environment (e.g., soil or water), and environmental factors can modulate parasite survival within the environment (Mouritsen and Poulin, 2003). Warm temperatures generally favour survival and infectivity of free-living stages, as in soil-transmitted helminths (Hernandez et al., 2013) or protozoa (Dubey, 2010), while excessively high temperatures can negatively affect parasite survival (van Dijk and Moran, 2008).

In the present study, we evaluate the influence of environmental factors on exposure of definitive host populations to *Toxoplasma gondii*, a generalist parasite with a complex life-cycle. This protozoan causes toxoplasmosis, one of the most prevalent zoonotic diseases of warm-blooded animals worldwide (Tenter et al., 2000). In humans, *T. gondii* infection can cause abortions or severe clinical symptoms in foetuses, neonates and immunocompromised individuals (Dubey, 2010). Due to the limited availability of effective treatment for humans (Gilbert, 2009), prevention of the infection is identified as a key method for reducing the disease burden (Kijlstra and Jongert, 2008). *T. gondii* has a complex life-cycle that involves both intermediate (mammals and birds) and definitive hosts (felids). Hosts are infected through the ingestion of either meat that contains bradyzoites or of oocysts present in the soil or water (Dubey, 2010). The domestic cat (*Felis silvestris catus*) and European wildcat (*Felis silvestris silvestris*) are the principal definitive hosts in Western Europe (Dubey, 2010). Most cats mainly excrete oocysts in the days or weeks after primary infection, although immunosuppression or chronic infections may lead to extended or repeated excretion (Dubey, 2010). For humans, oocysts are both a direct source of infection through contact with soil, water or vegetables and the main source of infection for meat producing animals. Although contamination with oocysts is a key issue in the epidemiology and prevention of *T. gondii* infection, its determinants are poorly understood.

*T. gondii* seroprevalence in its definitive hosts is known to be highly variable both spatially (Dubey et al., 2002; Silva et al., 2002; Afonso et al., 2010) and temporally (Salant and Spira, 2004; Afonso et al., 2010) but few data are available on the ecological determinants of this variability. Afonso et al. (2006, 2010) showed that seroprevalence in domestic cats was highest during years with high temperatures and/or high levels of rainfall, and the recent use of global climatic indices, such as the North Atlantic Oscillation winter index (NAO), in studies of parasite transmission provides an opportunity for studies of this temporal variability on a large geographical scale (Stenseth et al., 2002a; Mouritsen and Poulin, 2003). Landscape composition is another environmental factor that could also explain prevalence variability. For instance, farms used for agricultural production have been increasingly cited as suitable habitats for parasite transmission for the following reasons: (i) host concentration within a relatively small area (Patz et al., 2000); (ii) association with open landscapes where contacts between domestic and wild animals are frequent (Patz et al., 2000; Rosenthal, 2009); (iii) creation of moist and shaded microenvironments favourable to the long-term survival of free-living stage parasites; and (iv) increase in parasite dissemination via soil disruption by livestock animals (Lehmann et al., 2003).

The present study aimed to provide new insights into the determinants of the spatiotemporal variability in the seroprevalence of *T. gondii* in its definitive hosts, with a focus on landscape and climate. This variability was explored among communes during an 11-year collection of domestic cats, wildcats and their hybrids in Central and Eastern France.

## 2 Materials and methods

### 2.1 Sampling and serological analysis

**Fig. 1:**
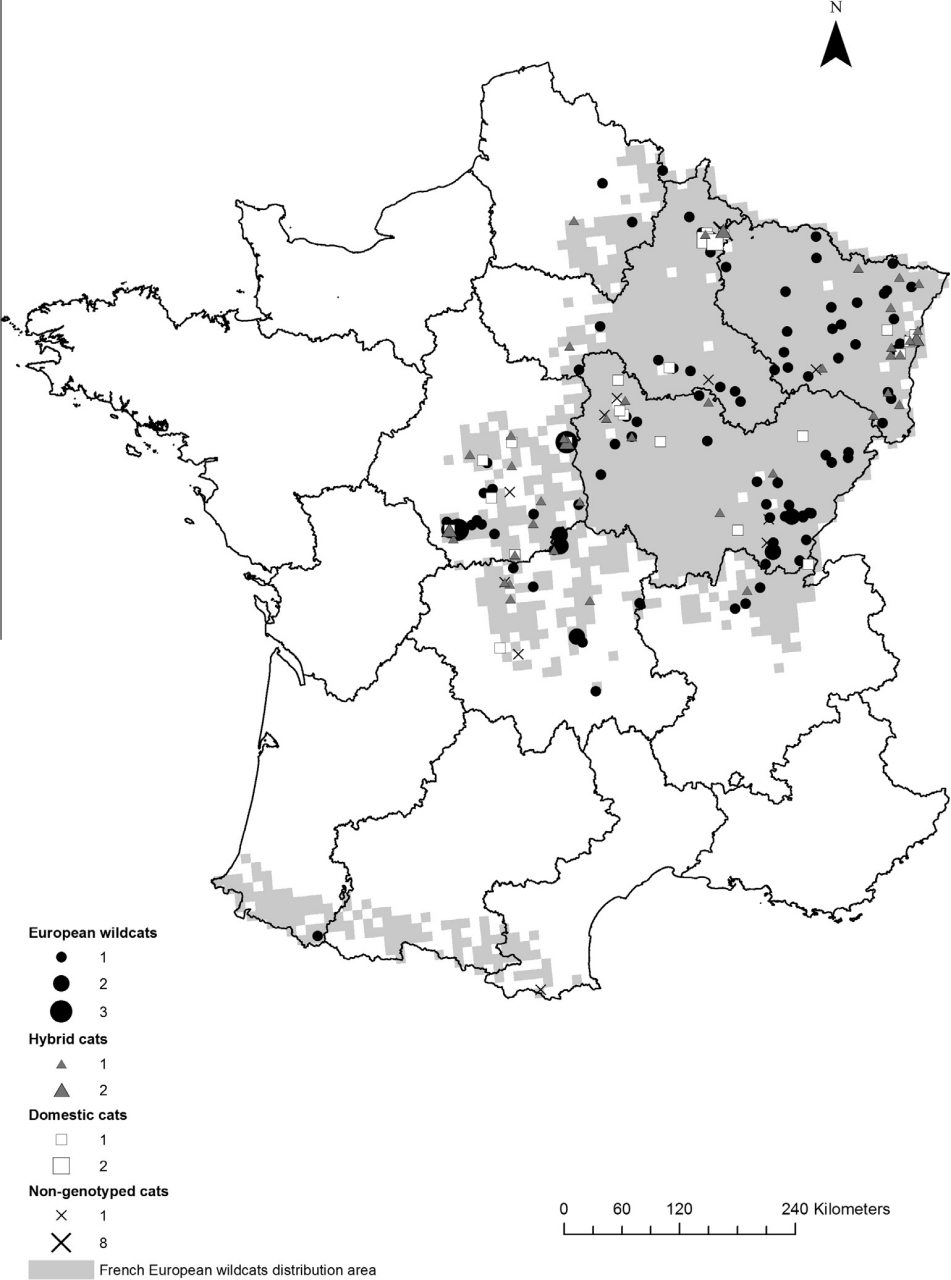
European wildcat (*Felis s. silvestris*) distribution in France (grey area; Léger et al., 2008; Say et al., 2012), and locations of samples from domestic cats (*Felis s. catus*), wildcats and their hybrids. Cat types are represented by different symbols (see the bottom left of the map). One location might correspond to several individuals (1, 2, 3, or 8), the size of the dot being proportional to the number (indicated at the right of the symbols) of individuals collected in each commune.

Between 1996 and 2006, 195 dead cats were collected from roads by the officers of the French Game and Wildlife Office (Office National de la Chasse et de la Faune Sauvage, ONCFS) in all regions where the presence of cats with a wildcat phenotype was suspected or confirmed (Central and Eastern France, see Léger et al., 2008; Fig. 1). In France, the domestic cat and the European wildcat are sympatric throughout the wildcat range, as are hybrids (Say et al., 2012). Additional samples were collected from 15 live-trapped cats in Eastern France between 2003 and 2006 (see Germain et al., 2008).

Animals found dead were necropsied in the laboratory (O’Brien et al., 2009; Say et al., 2012). Hair and blood samples were taken for genetic and serological analysis, respectively. Live-trapped cats were anaesthetised before sampling. Blood samples were centrifuged and the serum stored at −20 °C before use. Sera were tested for anti-*T. gondii* IgG antibodies with the modified agglutination test (Dubey and Desmonts, 1987). The serum (firstly diluted 1:3 using phosphate buffer salin (PBS) pH 7.2) was diluted 1:2 with 0.02 M dithiotreitol (DTT). The diluted serum (0.05 mL) was then placed in a round-bottom well of a microtiter plate and serial twofold dilutions were made in 0.025 mL of 0.02 M DTT. The antigen (whole killed *T. gondii* tachyzoites prepared at the laboratory of Parasitology, Reims, France) was then diluted (1:15 or 1: 20, depending on the batch of antigen) using the alkaline buffer BABS (Biomérieux, Marcy-L’Étoile, France) and 0.025 mL was added to each well. The plate was sealed with cellophane and kept overnight at room temperature. Positive and negative serum controls were included in all tests. Individuals were considered positive for anti-*T. gondii* antibodies if the titer was ⩾1:48 (Afonso et al., 2006).

### 2.2 Individual characteristics of cats

Individuals cats were weighed and sexed (variable *gender*), and the tarsus length was measured. The age class (variable *age*) was defined for all individuals. Those whose teeth were not fully erupted and/or were small in size (tarsus length <10 cm) were considered to be less than 1 year old and were classified as “juveniles”. The remaining cats were classified as “adults”. Cat body condition (variable *body condition*) was assessed as the residual of the linear regression of bodyweight versus tarsus length after both measures were converted into natural logarithms (Jakob et al., 1996). The body condition index was estimated separately for juveniles and adult individuals. The cat type (variable *type*: domestic cats, wildcats, and hybrids) was determined according to previously described molecular and analytical protocols (O’Brien et al., 2009; Say et al., 2012; see Appendix 1 in Supplementary data).

### 2.3 Investigation of spatial variation in *T. gondii* seroprevalence in relation to local environmental factors

*T. gondii* seroprevalence was expected to correlate positively with foci of human habitation because in rural areas these (farms and villages) are generally related to high local densities of domestic cats that might coincide with high local densities of intermediate hosts such as commensal rodents (Turner and Bateson, 2000). *T. gondii* seroprevalence was also expected to correlate positively with grasslands or forests where small mammals are generally present at high densities and where the three types of cats regularly hunt or defecate (Turner and Bateson, 2000; Biró et al., 2005). On the opposite, *T. gondii* seroprevalence was expected to correlate negatively with crops where small mammal abundance and diversity are generally lower than in grassland and forest (Butet et al., 2006; Heroldová et al., 2007). Besides landscape composition, several studies showed that livestock production farms can serve as *T. gondii* reservoirs in rural environments and can be hot spots for domestic cats and other small and large mammals (Smith et al., 1992; Dubey et al., 1995; Weigel et al., 1995, 1999; Lehmann et al., 2003; Richomme et al., 2010; Gilot-Fromont et al., 2012; Langlais et al., 2012). Thus, *T. gondii* infection was expected to be particularly frequent in communes with the highest farm densities.

Cats were assigned to the commune where they were trapped or collected on roads. The communes correspond to French administrative divisions, which consist of at least one village and the surrounding landscape. Commune-scale was chosen for the analysis because it was the smallest unit for which details of landscape composition and agricultural activities were available. In the present study, commune size ranged from 191 to 14,000 ha; these data were from the National Institute of Statistics and Economic Studies (http://www.insee.fr/). Each commune in which at least one cat was collected was characterised by its local landscape composition, farm density and local climate. Landscape composition in these communes was assessed with the CORINE Land Cover (CLC) France databases (25 ha resolution) that were produced by the European Environmental Agency (http://www.stats.environnement.developpement-durable.gouv.fr/clc/CORINE_Land_Cover_-_Saisie_Demande.jsp, accessed June, 2012) and the geographical information system ArcGIS 10 (ESRI; http://www.esrifrance.fr, accessed June, 2012). Land cover data from the CLC 2000 database were applied to cats collected from 1996 to 2000, whereas data from CLC 2006 were used for cats collected from 2001 to 2006. Land cover layers were grouped according to the CLC typology (see http://www.statistiques.developpement-durable.gouv.fr/donnees-ligne/t/nomenclature.html) as follows: (i) artificial surfaces included urban, industrial and commercial areas; (ii) cultivated agricultural areas included arable lands and permanent crops; (iii) grassed farmland areas included permanent grasslands, pastures, and heterogeneous agricultural areas; and (iv) forests, scrubs and herbaceous vegetation associations. Four variables (*foci of human habitation*, *crops*, *grasslands*, *forests*) were defined as the proportion of these four land cover classes in the communes to which the cats were assigned. The numbers of farms per commune were obtained from the French Ministry of Agriculture and Fisheries (http://www.agreste.agriculture.gouv.fr/). Farms were defined as all agricultural operations that produced either livestock and/or vegetables on at least one hectare of agricultural land. Farm density within a commune (variable *farms*) was then assessed as the number of farms divided by the area of the commune (km^2^).

Finally, we expected that seroprevalence in the three types of cats might be related to the local climate and would be the highest in the communes characterised by the highest temperatures and rainfall, as previously observed by Afonso et al. (2006, 2010). The climate of each commune was characterised according to the mean temperature of the coldest month of the year (*temperature*) and the mean annual rainfall (*rainfall*) over the past 30 years. These two meteorological variables are commonly used to describe the climate harshness for terrestrial species. The mean over 30 years (1982–2012) was used to adequately summarize the climatic conditions in a given commune without any influence of between-year variation. Meteorological conditions were obtained from MétéoFrance (http://www.meteofrance.com). The overall study area is characterized by a temperate climate with mean temperatures of the coldest month ranging from −2 °C to 3 °C, and annual rainfall ranging from 600 to 1 976 mm.

### 2.4 Investigation of the temporal variations in *T. gondii* seroprevalence over a large spatial area in relation to global climatic index

*The North Atlantic Oscillation winter index* was used to summarise the annual meteorological variations in the entire study area in order to analyse year-to-year variations in seroprevalence. This large-scale index measures the differences in pressure levels between Iceland and the Azores and thus provides a single variable with which to summarise interannual differences between several weather variables over a large geographic area, including temperature, wind speed and direction, as well as precipitation (Hurrell, 1995). The NAO winter index covers the period from December to March. In Western Europe, high index values are associated with high winter temperatures and high levels of precipitation (Hurrell, 1995). Each twelve month period of the study was divided into two: 21 December to 20 March, and 21 March to 20 December. Using this timetable, the value of the NAO winter index was recorded for the winter preceding the sampling of each cat. For example, for cats sampled between 21 December, 2000 and 20 March, 2001, the 1999–2000 winter was used, and for cats sampled between 21 March and 20 December, 2001, the 2000–2001 winter was used. The variable NAO was attributed differently for juveniles and adults. For juvenile cats the NAO winter index for one previous year was used, and for adult cats the mean NAO index for two and three previous winters. This was done because as *T. gondii* antibodies are lifelong, seropositivity could be related to a seroconversion that occurred several years ago. Since the precise age of cats is unknown in our dataset, we considered the median age of 2–3 years old estimated in previous studies conducted in owned or stray domestic cats living in rural or non anthropized areas (Afonso et al., 2007, 2010). We used NAO winter index values from the National Center for Atmospheric Research (NCAR, Boulder, Colorado, USA) are available online (http://www.cgd.ucar.edu/cas/jhurrell/indices.html, accessed June, 2013).

### 2.5 Statistical analysis

A logistic regression was used to relate the logit of the probability of infection for a cat to the predictor variables. Because the variable *NAO* was calculated differently for juveniles and adults, analyses were performed by considering two separate datasets according to age class. A forward selection was used to build the final equation; only some predictor variables were included per model to avoid confusion and over-parameterisation (see Appendix 2 in Supplementary data). At each analysis step, a series of models were built and compared according to the procedure described below. Individual characteristics (*gender*, *type*, and *body condition*) were considered in the logistic equation, and all models that included one or two variables such as *gender*, *type* and *body condition* with one two-order interaction were compared. Finally, the variables expected to explain the spatiotemporal variability of *T. gondii* seropositivity in cats (*foci of human habitation*, *crops*, *grasslands*, *forests*, *farms*, *temperature*, *rainfall*, *year* (included as a categorical variable), and *NAO*) were added to the model to test them once the individual characteristics were taken into account. Again, we compared all possible models, including the variables selected during the previous step and one or two supplementary variables, and their interactions.

The models were compared according to the Akaike’s Information Criterion, corrected for small sample size (AICc; Burnham and Anderson, 2001). AICc differences between the best model and all other considered models (Δ*_i_* = difference between AICc and the lowest AICc value) were calculated to determine the relative ranking of each possible model. The model with the lowest AICc represented the best compromise between the residual deviance and number of parameters (Burnham and Anderson, 2001). When Δ*_i_* < 2, the most parsimonious model (i.e., that with the fewest parameters) was selected. Odds-ratios and 95% confidence intervals (CIs) were used to measure the strength of association between each variable and the probability of infection while controlling for other variables. The overall fit of the final logistic equation was assessed with the Hosmer–Le Cessie test (Hosmer et al., 1997). All statistical procedures were performed with R 2.14.0 software (R Development Core Team, 2012, Vienna, Austria).

## 3 Results

**Fig. 2:**
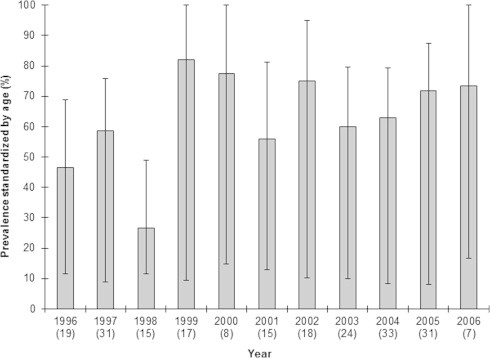
Interannual variations in *Toxoplasma gondii* seroprevalence in the three types of cats standardised by age (bars) during the study period. Line segments represent the 95% confidence intervals for seroprevalence, and the numbers in brackets indicate the sample sizes. Wildcats (*Felis s. silvestris*), domestic cats (*Felis s. catus*) and hybrids are pooled.

Overall, 210 cats were collected dead (*n* = 195) or live-trapped (*n* = 15) in 160 communes of Central and Eastern France (Fig. 1, Table 1). Among the cats, 44 were juveniles and 166 were adults; the sample included 29 domestic cats, 112 wildcats, 47 hybrids and 22 ungenotyped individuals. The age structure did not differ between the three types of cats (*χ*^2^ = 0.35, *df* = 2, *P* = 0.837); 17–22% of the collected individuals were juveniles and 78–83% were adults. The crude seroprevalence in the two age classes was estimated to be 45.5% in juveniles (20/44; 95% CI: 31.2–59.9) and 70.5% in adults (117/166; 95% CI: 63.1–76.9), and was significantly higher in adults than in juveniles (*χ*^2^ = 8.50, *df* = 1, *P* = 0.003). Of the 207 individuals for which gender was determined, 82 were females and 125 were males. The crude seroprevalence did not differ with gender (*χ*^2^ = 1.14, *df* = 1, *P* = 0.287) and was 59.8% in females (49/82; 95% CI: 48.9–69.7) and 68.0% in males (85/125; 95% CI: 59.4–75.5). Among the 210 cats, 137 were positive for *T. gondii*, and thus the crude overall seroprevalence was 65.2% (95% CI: 58.6–71.4). The crude seroprevalence did not differ among the three types of cats (*χ*^2^ = 0.03, *df* = 2, *P* = 0.985); seroprevalence was estimated to be 65.5% in domestic cats (19/29; 95% CI: 47.3–80.1), 65.9% in hybrids (31/47; 95% CI: 51.7–77.8), and 67.0% in wildcats (75/112; 95% CI: 57.8–75.0). During the study period (1996–2006), the observed seroprevalence was variable over time; the age-standardised seroprevalence ranged from a minimum value of 26.7%, recorded in 1998, to a maximum value of 82.4%, recorded in 1999 (Fig. 2).

**Fig. 3:**
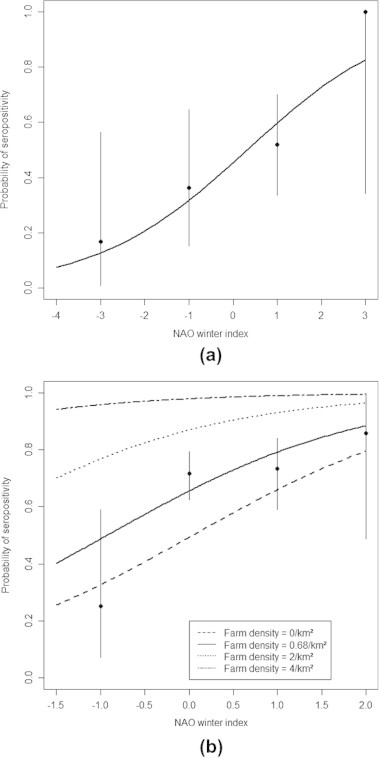
Predicted relationships between NAO winter index and the probability of seropositivity for *Toxoplasma gondii* in all cats sampled; (a) juveniles and (b) adults. Points represent the observed seoprevalence values with 95% confidence intervals as whiskers. A median farm density (0.68 farm/km^2^) was used to calculate the predictions (full line). Minimal farm density (0 farm/km^2^) observed among sampled communes was used to calculate the minimal predictions of the model (dotted lines). High values of farm density (2 and 4 farms/km^2^) were used to calculate the maximal predictions of the model (dashed and dotted-dashed lines).

The final logistic equation used to relate *T. gondii* seropositivity in juveniles to the predictor variables included only the effect of *NAO* (Table 2; see details on model selection in Appendix 2, Supplementary data). The probability of infection was related to large-scale meteorological fluctuations; seropositivity correlated positively with the NAO winter index (ΔAICc = 5.7). The probability of seropositivity increased 1.8-fold (95% CI: 1.1–2.9) with each increase of one NAO winter index unit (Fig. 3a).

Concerning adults, the final logistic equation included the effects of *farms* and *NAO* (Table 3; see details on model selection in Appendix 2, Supplementary data). Farm density strongly influenced *T. gondii* seropositivity in the three types of cats (ΔAICc = 4.9). The probability of *T. gondii* seropositivity increased 2.6-fold (95% CI: 1.1–6.4) with each increase of 1 farm/km^2^ (Fig. 3b). The farm density per commune ranged from 0.1 to 6.9 farms/km^2^ (mean = 0.86, median = 0.68); however, communes with farm densities >2 farms/km^2^ were rare in this dataset (3.8% of the 160 communes). Finally, the probability of infection was related to large-scale meteorological fluctuations; seropositivity correlated positively with the NAO winter index (ΔAICc = 2.2). NAO winter index values retained for adults were the mean of the three winters preceding the sampling. As expected, cats were more often seropositive when *NAO* was high. The probability of seropositivity increased 2.0-fold (95% CI: 1.1–4.0) with each increase of one NAO winter index unit.

All variables and two-order interactions including individual characteristics (*gender*, *type*, and *body condition*), landscape composition (*foci of human habitation*, *crops*, *grasslands*, *forests*), and meteorological conditions (*temperature*, *rainfall*) were not retained in the two final logistic models (0.1 < ΔAICc < 1.9, Appendix 2). The variable *NAO* was not retained in the model including adult cats when the mean of NAO winter index of the two winters preceding the collect of cats was considered (ΔAICc = 1.5). The Hosmer–Le Cessie goodness-of-fit test showed a good fit of the final selected model for juveniles (*P* = 0.467) and for adults (*P* = 0.784).

## 4 Discussion

This study, which was conducted in rural France, is the first to simultaneously estimate seroprevalences for *T. gondii* infection in the two main definitive hosts of this parasite (domestic cat and wildcat) and in their hybrids, as well as the first to relate these seroprevalence values to ecological descriptors. The levels of *T. gondii* seropositivity in the cats were 65.5% in domestic cats, 67.0% in European wildcats and 65.9% in their hybrids. These values might be biased by the fact that most animals sampled were road-kills, and that the search effort likely varied with location. Road-killed cats might be weakened, possibly partly due to *T. gondii*, and this bias might result in an overestimation of seropositivity in these cats. Furthermore, the autolysis of carcasses may lead to the degradation of proteins resulting in false negative serological results. In future studies, possible improvements in the sampling scheme include comparing individuals found dead and live-trapped in comparable areas (which was not the case here), and assessing the level of autolysis to test for its possible effect on the result of serological assay. However, despite these potential biases, the seroprevalences of *T. gondii* antibodies in cat populations did not vary with the type of cat and were comparable to values generally observed in other studies of domestic cats in rural environments (e.g., Tenter et al., 2000; Dubey and Jones, 2008).

The ingestion of one *T. gondii*-infected prey animal is sufficient to infect a cat (Dubey, 2010), and the seroprevalence of *T. gondii* in domestic cat populations depends on the number of prey ingested per cat per year (Lélu et al., 2010). The high levels of infection in the rural domestic cat population might thus be related to the frequent consumption of infected intermediate hosts (Tenter et al., 2000) because most rural free-roaming cats are known to be hunters even if fed daily (Tschanz et al., 2011). Wildcats might consume a greater number of intermediate hosts (small mammals) than domestic cats or hybrids (Biró et al., 2005; Germain et al., 2009), but domestic cats could prey on infected rodents around farms (Lehmann et al., 2003).

Adults were significantly more often positive (70.5%) than juveniles (45.5%). Because *T. gondii* antibodies persist for a lifetime (Afonso et al., 2006; Dubey, 2010) and vertical transmission is not a usual transmission route in cats (Dubey and Hoover, 1977; Afonso et al., 2006), adults were expected to have a higher seroprevalence of *T. gondii* positivity than juveniles. Furthermore, no relationship was detected between the *T. gondii* serological status in cats and land cover variables in the two age classes. In contrast, we showed that antibody prevalence was positively related to farm density in adults. Our approach was similar to that of Richomme et al. (2010), who showed that *T. gondii* seroprevalence in the wild boar (*Sus scrofa*) was related to farm densities within sampling communes. The commune, as a scale of investigation, does not have any significance for the ecology of the three types of cats (as home range size estimated from radiocollared individuals ranged from 2 to 220 ha for domestic cats and from 122 to 404 ha for wildcats (Germain et al., 2008), the range of one cat can overlap two communes). We assumed that farm densities within the communes reflected local agricultural management and therefore the importance of cat exposure to farm surroundings that are potential hyperendemic areas. Because farms are favourable areas for the presence of domestic cats (Warner, 1985; Turner and Bateson, 2000), wildlife (including wildcats and hybrids) that hunt in the vicinity of farms could be exposed to *T. gondii* oocysts that are present in the environment, or to infected intermediate hosts. These results suggest that *T. gondii* exposure may be more affected by the presence of highly localised areas supportive of parasite transmission (hyperendemic areas), for example farm building, than by a particular type of land cover. However, in our dataset, farm density correlated positively with grassed farmland areas (permanent grasslands and pastures; *R*^2^ = 0.13; data not shown), negatively with semi-natural areas (forests, scrub, and herbaceous vegetation; *R*^2^ = −0.30), and did not correlate with cultivated agricultural areas (arable lands and permanent crops; *R*^2^ = 0.06). Thus, a partial relationship between landscape components and *T. gondii* exposure might not be totally excluded and should be explored further. The proximity of farm buildings, instead of being a causal factor per se, may be associated with the epidemiology because of a particular landscape or specific contact patterns, all elements acting together to increase the risk of parasite transmission. For example, Daszak et al. (2006) found that orchard planting around pig farms increased interactions between fruit bats and pigs, thus influencing the transmission of the Nipah virus. Such a high geographical resolution approach could be used in studies of relationships between landscapes and *T. gondii* infection; however, map resolutions should be precise enough to clearly delimit the land cover around farms, which is not true of the geographical information currently available at the commune scale that was used for this study.

The variations of seroprevalence in our dataset correlated with large-scale meteorological variations. Because *T. gondii* antibodies are life-long, old, seropositive individuals may have been infected long before the study period, in which case their serological status should not depend on the conditions in the years of the study. As a consequence, at the population level, the correlation between yearly conditions and seroprevalence should be attenuated when the proportion of old individuals is high. Thus, relationships between meteorological parameters and animal serological status are likely detected when the turnover of individuals is high (due to dispersal or mortality) or when the influence of meteorological conditions is strong. However, seroprevalence in the three types of cats in the current study was highly related to the NAO winter index, as cats were more often positive for *T. gondii* when winter temperatures and rainfall were high during the previous years.

As predicted from the long-term persistence of antibodies, the seropositivity in juveniles was related to the mean NAO during the previous winter, while in adults it was related to the mean value over the three preceding winters. Relationships between infection rates of toxoplasmosis and meteorological variations have been observed or hypothesized in humans (Hubálek, 2005; Sukthana, 2006; Berger et al., 2008) as well as in animals (Almeria et al., 2004 for the rabbit *Oryctolagus cunniculus*; Gamarra et al., 2008 for the roe deer *Capreolus capreolus*; Afonso et al., 2010 in domestic cats), suggesting that, beside the infection in cats, the dynamics of the whole life cycle of *T. gondii* may be affected by meteorological conditions. These studies all concluded that high levels of rainfall increased prevalence because *T. gondii* oocysts were highly likely to survive during wet periods (Frenkel et al., 1975). Other effects of meteorological conditions can be suspected, such as influences on primary production (i.e. synthesis of organic compounds through photosynthesis or chemosynthesis) and rodent host population dynamics. These large-scale variations have complex interpretations, as they are linked to many aspects of disease transmission, yet are easy to measure and could thus constitute helpful risk indicators. One interesting result in the current study is that the local climate did not provide relevant information once the NAO winter index was taken into account, suggesting that spatio-temporal variations of seroprevalence are strongly driven by the global climate which determines temporal dynamics, while spatial variability is more related to landscape (farm density) than to any climatic factor. However, this study took place in Central and Eastern France, where the local climate might not vary much between communes. The spatiotemporal variations of *T. gondii* seroprevalence in definitive hosts might also vary at a larger scale between study areas where local climates contrast, and might also influence seroprevalence variability, as suggested in Afonso et al. (2010). Nevertheless, the NAO winter index has already been related to toxoplasmosis incidence in humans during a 38-year study period in the Czech Republic (Hubálek, 2005) and was interpreted as the result of an increase of population densities of intermediate hosts, then affecting the risk of infection in cats. These results reinforce the hypothesis of a general effect of meteorological variations on parasite transmission. One way to explore these results would be an investigation of the relationship between large-scale index values (NAO, El Niño) and primary productivity that influences small mammal abundance (Stenseth et al., 2002b); for example, the Normalised Difference Vegetation Index (NDVI) database could be used in a study of seroconversion data, which represent the number of individuals who seroconverted during a defined period of time.

Our results suggest that areas with high farm density and years with high NAO values are, respectively, high-risk places and high-risk periods for *T. gondii* infection in the three types of cats and thus for environmental contamination (oocyst spread in the soil or water). Moreover, the presence of farm buildings and the occurrence of rainy winters and mild winter temperatures should favour oocyst survival. These results remain to be explored, for example through an analysis of the spatiotemporal variations of environmental contamination. These elements must be considered in human risk analyses in order to make recommendations to improve prevention.

## Figures and Tables

**Table 1:** Main characteristics of the sample by type of cat.

			Domestic cats	Hybrids	Wildcats	Ungenotyped	Total
Live-trapped	Juveniles	♀	–	–	–	–	–
		♂	–	–	1	–	1
		nm	–	–	–	–	–
	Adults	♀	2	1	–	4	7
		♂	1	–	2	4	7
		nm	–	–	–	–	–

Road-killed	Juveniles	♀	4	6	8	2	20
		♂	1	3	16	2	22
		nm	–	–	–	1	1
	Adults	♀	9	10	33	3	55
		♂	12	26	51	6	95
		nm	–	1	1	0	2

Total			29	47	112	22	210

**Table 2:** Variables associated with seropositivity for *Toxoplasma gondii* in juvenile domestic cats (*Felis s. catus*), European wildcats (*Felis s. silvestris*) and their hybrids, and parameters of the final logistic equation.

Variable	*β*	SE*_β_*	Adjusted odds ratio	95% confidence interval	*P*-value (Wald test)
Intercept	−0.19	0.33	–	–	0.574
NAO winter index	0.58	0.24	1.8	[1.1; 2.9]	0.017

**Table 3:** Variables associated with seropositivity for *Toxoplasma gondii* in adult domestic cats (*Felis s. catus*), European wildcats (*Felis s. silvestris*) and their hybrids, and parameters of the final logistic equation.

Variable	*β*	SE*_β_*	Adjusted odds ratio	95% confidence interval	*P*-value (Wald test)
Intercept	−0.03	0.37	–	–	0.940
NAO winter index	0.69	0.35	2.0	[1.1; 4.0]	0.048
Farm density	0.96	0.46	2.6	[1.1; 6.4]	0.035

## Appendix Supplementary data

Supplementary data 1 This document contains ‘Appendix 1: Protocol for the identification of the genetic type of cats’ and ‘Appendix 2: Binomial model comparisons of the probability to be seropositive for *Toxoplasma gondii* related to predictor variables’.

## References

[b0005] Afonso E., Thulliez P., Gilot-Fromont E. (2006). Transmission of *Toxoplasma gondii* in an urban population of domestic cats (*Felis catus*). Int. J. Parasitol..

[b0010] Afonso E., Thulliez P., Pontier D., Gilot-Fromont E. (2007). Toxoplasmosis in prey species and consequences on prevalence in cats: not all preys are equal. Parasitology.

[b0015] Afonso E., Thulliez P., Gilot-Fromont E. (2010). Local meteorological conditions, dynamics of seroconversion to *Toxoplasma gondii* in cats (*Felis catus*) and oocyst burden in a rural environment. Epidemiol. Infect..

[b0020] Almeria S., Calvete C., Pagés A., Gauss C.B.L., Dubey J.P. (2004). Factors affecting the seroprevalence of *Toxoplasma gondii* infection in wild rabbits (*Oryctolagus cuniculus*) from Spain. Vet. Parasitol..

[b0025] Berger F., Goulet V., Le Strat Y., Desenclos J.-C. (2008). Toxoplasmose chez les femmes enceintes en France: évolution de la séroprévalence et de l’incidence et facteurs associés, 1995–2003. Bulletin Epidemiologique Hebdomadaire.

[b0030] Biró Z.S., Lanszki J., Szemethy L., Heltai M., Randi E. (2005). Feeding habits of feral domestic cats (*Felis catus*), wild cats (*Felis silvestris*) and their hybrids: trophic niche overlap among cat groups in Hungary. J. Zool..

[b0035] Burnham K.P., Anderson D.R., McCullough D.R., Barrett R.H. (2001). Data-based selection of an appropriate model: the key to modern data analysis. Wildlife 2001, Populations.

[b0040] Butet A., Paillat G., Delettre Y. (2006). Seasonal changes in small mammals assemblages from field boundaries in an agricultural landscape of western France. Agric. Ecosyst. Environ..

[b0045] Daszak P., Cunningham A.A., Hyatt A.D. (2001). Anthropogenic environmental change and the emergence of infectious diseases in wildlife. Acta Trop..

[b0050] Daszak P., Plowright R., Epstein J.H., Pulliam J., Abdul Rahman S., Field H.E., Jamaluddin A., Sharifah S.H., Smith C.S., Olival K.J., Luby S., Halpin K., Hyatt A.D., Cunningham A.A., Henipavirus Ecology Research Group, Collinge S., Ray C. (2006). The emergence of Nipah and Hendra virus: pathogen dynamics across a wildlife–livestock–human continuum. Disease Ecology: Community Structure and Pathogen Dynamics.

[b0055] Dubey J.P. (2010). Toxoplasmosis of Animals and Humans.

[b0060] Dubey J.P., Desmonts G. (1987). Serological responses of equids fed *Toxoplasma gondii* oocysts. Equine Vet. J..

[b0065] Dubey J.P., Hoover E.A. (1977). Attempted transmission of *Toxoplasma gondii* infection from pregnant cats to their kittens. J. Am. Vet. Med. Assoc..

[b0070] Dubey J.P., Jones J.L. (2008). *Toxoplasma gondii* infection in humans and animals in the United States. Int. J. Parasitol..

[b0075] Dubey J.P., Weigel R.M., Siegel A.M., Thuilliez P., Kitron U.D., Mitchell M.A., Mannelli A., Mateus-Pinilla N.E., Shen S.K., Kwok O.C.H., Todd K.S. (1995). Sources and reservoirs of *Toxoplasma gondii* infection on 47 swine farms in Illinois. J. Parasitol..

[b0080] Dubey J.P., Saville W.J.A., Stanek J.F., Reed S.M. (2002). Prevalence of *Toxoplasma gondii* antibodies in domestic cats from rural Ohio. J. Parasitol..

[b0085] Frenkel J.K., Ruiz A., Chinchilla M. (1975). Soil survival of *Toxoplasma* oocysts in Kansas and Costa Rica. Am. J. Trop. Med. Hyg..

[b0090] Gamarra J.A., Cabezón O., Pabón M., Arnal M.C., Luco D.F., Dubey J.P., Gortázar C., Almeria S. (2008). Prevalence of antibodies against *Toxoplasma gondii* in Roe deer from Spain. Vet. Parasitol..

[b0095] Germain E., Benhamou S., Poulle M.-L. (2008). Spatio-temporal sharing between the European wildcat (*Felis silvestris*), the domestic cat (*Felis catus*), and their hybrids. J. Zool..

[b0100] Germain E., Ruette S., Poulle M.-L. (2009). Likeness between the food habits of European wildcats, domestic cats and their hybrids in France. Mamm. Biol..

[b0105] Gilbert R. (2009). Treatment for congenital toxoplasmosis: finding out what works. Mem. Inst. Oswaldo Cruz.

[b0110] Gilot-Fromont E., Lélu M., Dardé M.-L., Richomme C., Aubert D., Afonso E., Mercier A., Gotteland C., Villena I., Djaković Olgica Djurković (2012). The life cycle of *Toxoplasma gondii* in the natural environment. Toxoplasmosis – Recent Advances.

[b0115] Giraudoux P., Craig P.S., Delattre P., Bao G., Bartholomot B., Harraga S., Quéré J.P., Raoul F., Wang Y., Shi D., Vuitton D.A. (2003). Interactions between landscape changes and host communities can regulate *Echinococcus multilocularis* transmission. Parasitology.

[b0300] Hernandez A.D., Poole A., Cattadori I.M. (2013). Climate changes influence free-living stages of soil-transmitted parasites of European rabbits. Glob. Chang. Biol..

[b0125] Heroldová M., Bryja J., Zejda J., Tkadlec E. (2007). Structure and diversity of small mammal communities in agriculture landscape. Agric. Ecosyst. Environ..

[b0130] Hosmer D.W., Hosmer T., Le Cessie S., Lemeshow S. (1997). A comparison of goodness of fit tests for the logistic regression model. Stat. Med..

[b0135] Hubálek Z. (2005). North Atlantic weather oscillation and human infectious diseases in the Czech Republic, 1951–2003. Eur. J. Epidemiol..

[b0140] Hurrell J.W. (1995). Deacadal trends in the North Atlantic oscillation: regional temperatures and precipitation. Science.

[b0145] Jakob E.M., Marshall S.D., Uetz G.W. (1996). Estimating fitness: a comparison of body condition indices. Oikos.

[b0150] Kijlstra A., Jongert E. (2008). Control of the risk of human toxoplasmosis transmitted by meat. Int. J. Parasitol..

[b0155] Langlais M., Lélu M., Avenet C., Gilot-Fromont E. (2012). A simplified model system for *Toxoplasma gondii* spread within a heterogeneous environment. Nonlinear Dyn..

[b0160] Léger F., Stahl P., Ruette S., Wilhelm J.-L. (2008). La répartition du Chat forestier en France: évolutions récentes. Faune Sauvage.

[b0165] Lehmann T., Graham D.H., Dahl E., Sreekumar C., Launer F., Corn J.L., Gamble H.R., Dubey J.P. (2003). Transmission dynamics of *Toxoplasma gondii* on a pig farm. Infect. Genet. Evol..

[b0170] Lélu M., Langlais M., Poulle M.-L., Gilot-Fromont E. (2010). Transmission Dynamics of *Toxoplasma gondii* along a urban–rural gradient. Theor. Popul. Biol..

[b0175] Lidicker W.Z. (1995). Landscape Approaches in Mammalian Ecology and Conservation.

[b0180] Malo A.F., Lozano J., Huertas D.L., Virgos E. (2004). A change of diet from rodents to rabbits (*Oryctolagus cuniculus*). Is the wildcat (*Felis silvestris*) a specialist predator?. J. Zool..

[b0185] Mouritsen K.N., Poulin R. (2003). Parasitism, climate oscillations and the structure of natural communities. Oikos.

[b0190] O’Brien J., Devillard S., Say L., Vanthomme H., Léger F., Ruette S., Pontier D. (2009). Preserving genetic integrity in a hybridising world: are European Wildcats (*Felis silvestris silvestris*) in eastern France distinct from sympatric feral domestic cats?. Biodivers. Conserv..

[b0195] Ottersen G., Planque B., Belgrano A., Post E., Reid P.C., Stenseth N.C. (2001). Ecological effects of the North Atlantic oscillation. Oecologia.

[b0200] Patz J.A., Graczyk T.K., Geller N., Vittor A.Y. (2000). Effects of environmental change on emerging parastic diseases. Int. J. Parasitol..

[b0205] R Development Core Team (2012). R: A Language and Environment for Statistical Computing. http://www.R-project.org.

[b0210] Raoul F., Deplazes P., Rieffel D., Lambert J.-C., Giraudoux P. (2010). Predator dietary response to prey density variation and consequences for cestode transmission. Oecologia.

[b0215] Richomme C., Afonso E., Tolon V., Ducrot C., Halos L., Alliot A., Perret C., Thomas M., Boireau P., Gilot-Fromont E. (2010). Seroprevalence and factors associated with *Toxoplasma gondii* infection in wild boar (*Sus scrofa*) in a Mediterranean island. Epidemiol. Infect..

[b0220] Rosenthal B.M. (2009). How has agriculture influenced the geography and genetics of animal parasites?. Trends Parasitol..

[b0225] Salant H., Spira D.T. (2004). A cross-sectional survey of anti-*Toxoplasma gondii* antibodies in Jerusalem cats. Vet. Parasitol..

[b0230] Say L., Devillard S., Léger F., Pontier D., Ruette S. (2012). Distribution and spatial genetic structure of European wildcat in France. Anim. Conserv..

[b0235] Silva J.C.R., Gennari S.M., Ragozo A.M.A., Amajones V.R., Magnabosco C., Yai L.E.O., Ferreira-Neto J.S., Dubey J.P. (2002). Prevalence of *Toxoplasma gondii* antibodies in sera of domestic cats from Guarulhos and São Paulo, Brazil. J. Parasitol..

[b0240] Smith K.E., Zimmermann J.J., Patton S., Beran G.W., Hill H.T. (1992). The epidemiology of toxoplasmosis in Iowa swine farms with an emphasis on the roles of free-living mammals. Vet. Parasitol..

[b0245] Stenseth N.C., Chan K.-S., Tong K., Boonstra R., Boutin S., Krebs C.J., Post E., O’Donoghue M., Yoccoz N.G., Forchhammer M.C., Hurrell J.W. (1999). Common dynamic structure of Canada Lynx populations within three climatic regions. Science.

[b0250] Stenseth N.C., Mysterud A., Otterson G., Hurrel J.W., Chan K.S., Lima M. (2002). Ecological effects of climate fluctuations. Science.

[b0255] Stenseth N.C., Viljugrein H., Jedrzejewski W., Mysterud A., Pucek Z. (2002). Population dynamics of *Clethrionomys glareolus* and *Apodemus flavicollis*: seasonal components of density dependence and density independence. Acta Theriol..

[b0260] Sukthana Y. (2006). Toxoplasmosis: beyond animals to humans. Trends Parasitol..

[b0265] Tschanz B., Hegglin D., Gloor S., Bontadina F. (2011). Hunters and non-hunters: skewed predation rate by domestic cats in a rural village. Eur. J. Wildl. Res..

[b0270] Tenter A.M., Heckeroth A.R., Weiss L.M. (2000). *Toxoplasma gondii*: from animals to humans. Int. J. Parasitol..

[b0275] Turner D.C., Bateson P.B. (2000). The Domestic Cat. The Biology of its Behaviour.

[b0280] van Dijk J., Moran E.R. (2008). The influence of temperature on the development, hatching and survival of *Nematodirus battus* larvae. Parasitology.

[b0285] Warner R.E. (1985). Demography and movements of free-ranging domestic cats in rural areas Illinois. J. Wildl. Manage..

[b0290] Weigel R.M., Dubey J.P., Siegel A.M., Kitron U.D., Mannelli A., Mitchell M.A., Mateus-Pinilla N.E., Thuilliez P., Shen S.K., Kwok O.C.H., Todd K.S. (1995). Risk factors for transmission of *Toxoplasma gondii* on swine farms in Illinois. J. Parasitol..

[b0295] Weigel R.M., Dubey J.P., Dyer D., Siegel A.M. (1999). Risk factors for infection with *Toxoplasma gondii* for residents and workers on swine farms in Illinois. Am. J. Trop. Med. Hyg..

