# Supplementary material for: Environmental determinants of spatial and temporal variations in the transmission of Toxoplasma gondii in its definitive hosts
Source: Int J Parasitol Parasites Wildl. 2013 Sep 23;2:278–85. doi: 10.1016/j.ijppaw.2013.09.006 (PMC3862504; doi:10.1016/j.ijppaw.2013.09.006)
Supplement: Supplementary data 1 — This document contains ‘Appendix 1: Protocol for the identification of the genetic type of cats’ and ‘Appendix 2: Binomial model comparisons of the probability to be seropositive for Toxoplasma gondii related to predictor variables’. [file mmc1.docx]

**Appendix 1. Protocol for the identification of the genetic type of cats.**

The n=188 specimens with a genetic classification (variable *type*) used in the analysis constitute a sub-sample of a broader one which is made up of n=341 specimens. We used STRUCTURE (Pritchard *et al*. 2000) following exactly the methodology in O’Brien *et al.* (2009) and in Say *et al.* (2012) to identify domestic, hybrid and wildcat specimens. Genotypes of the 341 individuals (75 cats in addition to the 266 previously used in Say *et al.* 2012; 221 presumed wildcats, 62 presumed feral domestic cats and 62 unclassified cats *sensu* O’Brien *et al.* 2009) were thus available at 12 microsatellite loci.

*Data Analysis*

We used the program STRUCTURE v2.1 (Pritchard *et al.* 2000) to a) identify population substructure among the samples in terms of the number of K clusters and b) to assign the proportion of each individual’s genotype derived from each of these K clusters. STRUCTURE uses Bayesian Monte-Carlo Markov Chain sampling to identify the optimal number of clusters for a given multi-locus dataset by minimising departures from Hardy-Weinberg and linkage equilibrium expectations, without needing to identify population subunits *a priori*. We used 500,000 generations, of which the first 20,000 were discarded as burn-in, and applied the admixture model with correlated allele frequencies due to the possibility of hybrid individuals being present in the sample set. We simulated the dataset for K = 1 through to K = 6 and performed 20 STRUCTURE runs for each value of K. We then employed the method of Evanno *et al*. (2005) to assess the optimal value of K (*i.e*. the optimal number of clusters in the dataset).

Simultaneously, STRUCTURE calculates the proportion (*q_ik_*) of each genotype of individual samples that is derived from each of the K clusters. Individual samples can have membership in multiple clusters, but membership coefficients (*q_ik_* values) sum to unity across clusters. Thus, the values of *q_ik_* can be effectively used to identify first generation hybrid or admixed animals if their *q_ik_* value is split between different clusters.

*Power of admixture analysis and threshold determination*

The proportion of parental and admixed (hybrid) individuals in a given sample is very sensitive to the threshold value of *q_ik_* used to assign each individual in a given cluster (Vähä and Primmer 2006). Therefore, we assessed the power of admixture analysis on simulated genotypes to avoid false classification of admixed or parental individuals (Barilani *et al.* 2007, Oliveira *et al*. 2008). Two subsamples consisting of the 30 individuals showing the highest *q_ik_* values for the “wild” and “domestic” clusters respectively were created to generate samples for four classes (parental, F1 and F2 populations, and backcross hybrids) using the function *hybridize* of the R package *adegenet 1.3* (Jombart 2008, R Development Core Team 2012). By taking only the highest *q_ik_* values, we excluded possible admixed individuals from the parental populations used to simulate F1, F2 and backcross hybrids. From these two initial subsamples of wildcat and domestic parental populations, we simulated 100 genotypes of each of the four classes, replicated 10 times. The simulated genotypes were used to carry out admixture analysis with STRUCTURE with K=2 and using the admixture model with correlated allele frequencies and no *a priori* information on population structure. The proportion of simulated genotypes correctly assigned was used to assess the efficiency of the admixture analysis to detect the different classes of parental, and, F1, F2 and backcross admixed genotypes, and to choose reliable threshold *q_ik_* values to assign individuals into different genetic clusters.

*Results of the STRUCTURE analysis*

The results of analysis with STRUCTURE without *a priori* information on sample phenotypic classification are presented in Figure 1. The variation of the Ln P(D) values with the number of inferred clusters K (Figure 1a) indicated that K = 2 is the most likely number of clusters, which was confirmed by the Evanno *et al*. (2005) (Figure 1b).


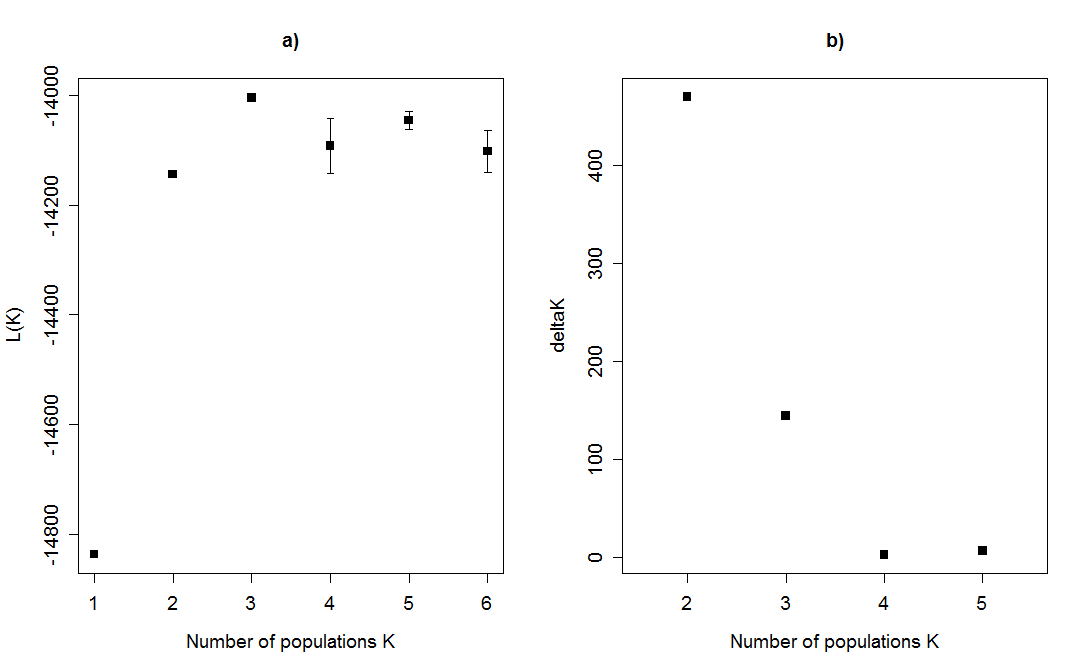


***Figure 1*** *a) Mean (± SD) of Ln P(D) over 20 Structure runs for successive K values. Variations of ΔK as calculated by Evanno et al. (2005) for successive K values*

We therefore estimated the proportion of membership of each predefined phenotypic group into these two genetic clusters. STRUCTURE differentiated well between presumed wildcats (“wildcat” cluster) and presumed domestic cats (“domestic” cluster); the unclassified cats being more evenly distributed between the two identified clusters (Table 1) suggesting that both wildcats, domestic and admixed cats coexist in our sample.

***Table 1*** *Genome partition within the two inferred clusters for the three predefined phenotypic groups*

|  | Cluster 1 “Domestic” | Cluster 2 “Wildcats” | Sample size |
| --- | --- | --- | --- |
| Presumed wilcat | 0.110 | 0.890 | 221 |
| Unclassified | 0.340 | 0.660 | 62 |
| Presumed domestic | 0.871 | 0.129 | 62 |

The simulation study provided important insights into the threshold values most appropriate for assigning individuals into the three groups (wildcat, admixed and domestic). Parental simulated genotypes and F1, F2, backcross admixed simulated genotypes were obtained from the 30 individuals having the higher *q_ik_* values for the “wild” cluster (i.e. *q_ik_* >0.981) and from the 30 individuals showing the lower *q_ik_* for the “wild” cluster (i.e. *q_ik_* <0.036) in the previous STRUCTURE admixture analysis.

***Table 1*** *Mean and range over 10 replicates of the simulation procedure for the maximum value of proportion of membership (q_ik_) of simulated hybrids (Wild X F1 backcross, F1, F2, Domestic X F1 backcross) in both wild and domestic parental clusters.*

|  | Wild parental cluster | Domestic parental cluster |
| --- | --- | --- |
| Wild X F1 backcross | 0.767 [0.978 – 0.991] | 0.233 [0.466 – 0.602] |
| F1 | 0.505 [0.641 – 0.822] | 0.496 [0.647 – 0.755] |
| F2 | 0.495 [0.762 – 0.869] | 0.502 [0.797 – 0.914] |
| Domestic X F1 backcross | 0.190 [0.458 – 0.748] | 0.810 [0.983 – 0.992] |

None of the simulated F1 and F2 individuals showed a membership proportion *q_ik_* greater than 0.822 and 0.869, respectively, for the “wildcat” parental cluster (Table 1). However, and as in O’Brien *et al*. (2009), our analysis did not allow us to distinguish between wildcat parent and wildcat X F1 backcross individuals, as the maximum value of membership proportion (*q_ik_*) for the “wildcat” cluster ranged from 0.978 to 0.991 over the 10 simulations (Table 1). Consequently, we define wildcats as all specimens having a membership proportion (*q_ik_*) for the “wildcat” cluster higher than 0.870, a threshold slightly lower than in O’Brien *et al*. (2009), albeit acknowledging that these wildcats might be either wild or backcrossed cats. Similarly, none of the simulated F1 and F2 individuals showed a membership proportion (*q_ik_*) higher than 0.755 and 0.914, respectively, for the “domestic” parental cluster (Table 1), and again domestic X F1 backcross individuals could not be distinguished from domestic parents (membership proportion *q_ik_* for the domestic cluster ranged from 0.983 to 0.992, Table 1). Therefore, the specimens defined as domestic cats were those having a membership proportion *q_ik_* for the “domestic” cluster greater than 0.914, again a threshold slightly lower than in O’Brien *et al*. (2009).

From this analysis, we extracted the genetic classification (domestic cat, hybrid, wildcat) for the n=188 cats (29 domestic cats, 47 hybrids, 112 wildcats) used in the epidemiological analysis.

References

Barilani M, Sfougaris A, Giannakopoulos A, Mucci N, Tabarroni C, Randi E (2007) Detecting introgressive hybridisation in rock partridge populations (*Alectoris graeca*) in Greece through Bayesian admixture analyses of multilocus genotypes. Conserv Genet 8:343–354.

Evanno G, Regnaut S, Goudet J (2005) Detecting the number of clusters of individuals using the software structure: a simulation study. Mol Ecol 14:2611–2620.

Jombart T (2008) *adegenet*: a R package for the multivariate analysis of genetic markers. Bioinformatics **24**:1403–1405.

O’Brien J, Devillard S, Say L, Vanthomme H, Leger F, Ruette S, Pontier D (2009) Preserving genetic integrity in a hybridising world: are European wildcats (*Felis silvestris silvestris*) in eastern France distinct from sympatric feral domestic cats? Biodiv Conserv 18:2351–2360.

Oliveira R, Godhino R, Pierpaoli M, Randi E, Ferrand N, Alves PC (2008) Molecular analysis of hybridization between wild and domestic cat (*Felis silvestris*) in Portugal: implication for conservation. Conserv Genet 9:1–11.

Say L, Devillard S, Leger F, Pontier D, Ruette S (2012) Distribution and spatial genetic structure of European wildcat in France. Anim Conserv **15**:18–27.

Pritchard J, Stephens M, Donnelly P (2000) Inference of population structure using multilocus genotype data. Genetics **155**:945–959.

R Development Core Team (2012). R: A language and environment for statistical computing. R Foundation for Statistical Computing, Vienna, Austria. http://www.R-project.org/

Vähä J-C, Primmer CR (2006) Efficiency of model-based Bayesian methods for detecting hybrid individuals under different hybridization scenarios and with different numbers of loci. Mol Ecol 15:63–72.

**Appendix 2. Binomial model comparisons of the probability to be seropositive for *Toxoplasma gondii* related to predictor variables.**

1. **Juveniles (N = 44)**

| **Model** | **LL** | **K** | **n/K** | **AICc** | **Δ_i_** | **w_ic_** |
| --- | --- | --- | --- | --- | --- | --- |
| a) Individual characteristics |  |  |  |  |  |  |
|  |  |  |  |  |  |  |
| *gender* | -29.5 | 2 | 22.0 | 63.3 | 1.0 | 0.4 |
| ***1*** | **-30.1** | **1** | **44.0** | **62.3** | **0.0** | **0.6** |
|  |  |  |  |  |  |  |
| *type* | -26.7 | 3 | 13.3 | 60.2 | 3.0 | 0.2 |
| ***1*** | **-27.5** | **1** | **44.0** | **57.2** | **0.0** | **0.8** |
|  |  |  |  |  |  |  |
| *body condition* | -28.7 | 2 | 21.0 | 61.7 | 2.2 | 0.2 |
| ***1*** | **-28.7** | **1** | **42.0** | **59.5** | **0.0** | **0.8** |
|  |  |  |  |  |  |  |
| *gender + type + gender:type* | -25.1 | 6 | 6.5 | 64.9 | 8.9 | 0.0 |
| *gender + type* | -25.7 | 4 | 9.8 | 60.6 | 2.0 | 0.1 |
| *gender* | -26.8 | 2 | 19.5 | 58.0 | 4.7 | 0.3 |
| ***1*** | **-26.9** | **1** | **39.0** | **55.9** | **0.0** | **0.7** |
|  |  |  |  |  |  |  |
| *gender + body condition + gender: body condition* | -26.9 | 4 | 10.3 | 62.9 | 4.5 | 0.1 |
| *gender + body condition* | -28.1 | 3 | 13.7 | 62.8 | 4.5 | 0.1 |
| *gender* | -28.1 | 2 | 20.5 | 60.5 | 2.2 | 0.2 |
| ***1*** | **-28.1** | **1** | **41.0** | **58.3** | **0.0** | **0.6** |
|  |  |  |  |  |  |  |
| *type + body condition + type: body condition* | -22.8 | 6 | 6.3 | 60.2 | 6.4 | 0.1 |
| *type + body condition* | -23.1 | 4 | 9.5 | 55.4 | 1.5 | 0.2 |
| *type* | -23.6 | 3 | 12.7 | 53.8 | 0.0 | 0.4 |
| ***1*** | **-26.1** | **1** | **38.0** | **54.4** | **0.6** | **0.3** |
|  |  |  |  |  |  |  |
|  |  |  |  |  |  |  |
| b) Environmental characteristics |  |  |  |  |  |  |
|  |  |  |  |  |  |  |
| *artificial* | -27.5 | 2 | 20.0 | 59.3 | 2.1 | 0.2 |
| ***1*** | **-27.5** | **1** | **40.0** | **57.2** | **0.0** | **0.8** |
|  |  |  |  |  |  |  |
| *crops* | -27.5 | 2 | 20.0 | 59.3 | 2.1 | 0.2 |
| *1* | **-27.5** | **1** | **40.0** | **57.2** | **0.0** | **0.8** |
|  |  |  |  |  |  |  |
| *grasslands* | -26.8 | 2 | 20.0 | 57.9 | 0.7 | 0.4 |
| ***1*** | **-27.5** | **1** | **40.0** | **57.2** | **0.0** | **0.6** |
|  |  |  |  |  |  |  |
| *forests* | -26.8 | 2 | 20.0 | 58.0 | 0.8 | 0.4 |
| ***1*** | **-27.5** | **1** | **40.0** | **57.2** | **0.0** | **0.6** |
|  |  |  |  |  |  |  |
| ***farms*** | **-26.7** | **2** | **22.0** | **57.6** | **0.0** | **0.9** |
| *1* | -30.1 | 1 | 44.0 | 62.3 | 4.7 | 0.1 |
| **Model** | **LL** | **K** | **n/K** | **AICc** | **Δ_i_** | **w_ic_** |
| *temperature + rainfall + temperature:rainfall* | -22.8 | 4 | 10.3 | 54.8 | 2.5 | 0.2 |
| ***temperature + rainfall*** | **-22.8** | **3** | **13.7** | **52.3** | **0.0** | **0.7** |
| *temperature* | -26.7 | 2 | 20.5 | 57.7 | 5.4 | 0.1 |
| *1* | -28.1 | 1 | 41.0 | 58.3 | 6.0 | 0.0 |
|  |  |  |  |  |  |  |
| *year* | -22.6 | 11 | 4.0 | 75.5 | 13.2 | 0.0 |
| ***1*** | **-30.1** | **1** | **44.0** | **62.3** | **0.0** | **1** |
|  |  |  |  |  |  |  |
| ***NAO*** | **-26.1** | **2** | **22.0** | **56.6** | **0.0** | **1.0** |
| *1* | -30.1 | 1 | 44.0 | 62.3 | 5.7 | 0.0 |
|  |  |  |  |  |  |  |
| *NAO + farms* | -23.0 | 3 | 13.3 | 52.7 | 1.5 | 0.3 |
| ***NAO*** | **-23.4** | **2** | **20.0** | **51.2** | **0.0** | **0.7** |
| *1* | -27.3 | 1 | 40.0 | 56.7 | 5.5 | 0.0 |
|  |  |  |  |  |  |  |
| *NAO + temperature + rainfall* | -23.4 | 4 | 10.3 | 56.8 | 4.1 | 0.0 |
| *NAO + temperature* | -23.5 | 3 | 13.7 | 54.1 | 1.4 | 0.2 |
| ***NAO*** | **-24.2** | **2** | **20.5** | **52.7** | **0.0** | **0.8** |
| *1* | -28.1 | 1 | 41.0 | 58.3 | 5.6 | 0.0 |
|  |  |  |  |  |  |  |
|  |  |  |  |  |  |  |
| c) Final logistic equation |  |  |  |  |  |  |
|  |  |  |  |  |  |  |
| ***NAO*** | **-26.1** | **2** | **22.0** | **56.6** | **0.0** | **1.0** |
| *1* | -30.1 | 1 | 44.0 | 62.3 | 5.7 | 0.0 |
|  |  |  |  |  |  |  |

LL, Maximized log-likelihood; K, Number of estimated parameters; n/K, number of observations/K; AICc, Akaike’s Information Criterion ; Δi, difference between AIC and the lowest AIC value; w_ic_, Akaike weight. Values in bold correspond to the selected models.

1. **Adults (N = 166)**

| **Model** | **LL** | **K** | **n/K** | **AICc** | **Δ_i_** | **w_ic_** |
| --- | --- | --- | --- | --- | --- | --- |
| a) Individual characteristics |  |  |  |  |  |  |
| *gender* | -99.3 | 2 | 82.0 | 202.6 | 0.6 | 0.4 |
| ***1*** | **-100.0** | **1** | **164.0** | **202.0** | **0.0** | **0.6** |
|  |  |  |  |  |  |  |
| *type* | -88.2 | 2 | 49.3 | 182.6 | 4.0 | 0.1 |
| ***1*** | **-88.3** | **1** | **148.0** | **178.6** | **0.0** | **0.9** |
|  |  |  |  |  |  |  |
| *body condition* | -86.8 | 2 | 72.5 | 177.6 | 1.0 | 0.4 |
| ***1*** | **-87.3** | **1** | **145.0** | **176.6** | **0.0** | **0.6** |
|  |  |  |  |  |  |  |
| *gender + type + gender:type* | -85.3 | 6 | 24.5 | 183.2 | 5.3 | 0.0 |
| *gender + type* | -87.0 | 4 | 36.8 | 182.3 | 4.4 | 0.1 |
| *gender* | -87.2 | 2 | 73.5 | 178.5 | 0.5 | 0.4 |
| ***1*** | **-87.9** | **1** | **147.0** | **177.9** | **0.0** | **0.5** |
|  |  |  |  |  |  |  |
| *gender + body condition + gender: body condition* | -85.3 | 4 | 36.0 | 178.9 | 3.0 | 0.2 |
| *gender + body condition* | -84.5 | 3 | 48.0 | 177.0 | 1.1 | 0.2 |
| *gender* | -86.5 | 2 | 72.0 | 177.0 | 1.1 | 0.2 |
| ***1*** | **-86.9** | **1** | **144.0** | **175.9** | **0.0** | **0.4** |
|  |  |  |  |  |  |  |
| *type + body condition + type: body condition* | -81.6 | 6 | 23.0 | 175.9 | 6.0 | 0.0 |
| *type + body condition* | -83.3 | 4 | 34.5 | 174.9 | 5.0 | 0.1 |
| *type* | -83.9 | 3 | 46.0 | 174.0 | 4.1 | 0.1 |
| ***1*** | **-84.0** | **1** | **138.0** | **169.9** | **0.0** | **0.8** |
|  |  |  |  |  |  |  |
| b) Environmental characteristics |  |  |  |  |  |  |
|  |  |  |  |  |  |  |
| *artificial* | -95.0 | 2 | 79.5 | 194.0 | 0.7 | 0.4 |
| ***1*** | **-95.6** | **1** | **159.0** | **193.3** | **0.0** | **0.6** |
|  |  |  |  |  |  |  |
| *crops* | -95.6 | 2 | 79.5 | 195.3 | 2.0 | 0.3 |
| ***1*** | **-95.6** | **1** | **159.0** | **193.3** | **0.0** | **0.7** |
|  |  |  |  |  |  |  |
| *grasslands* | -95.3 | 2 | 79.5 | 194.7 | 1.4 | 0.3 |
| ***1*** | **-95.6** | **1** | **159.0** | **193.3** | **0.0** | **0.7** |
|  |  |  |  |  |  |  |
| *forests* | -94.9 | 2 | 79.5 | 193.9 | 0.6 | 0.4 |
| ***1*** | **-95.6** | **1** | **159.0** | **193.3** | **0.0** | **0.6** |
|  |  |  |  |  |  |  |
| ***farms*** | **-88.5** | **2** | **75.5** | **181.1** | **0.0** | **0.9** |
| *1* | -92.0 | 1 | 151.0 | 186.0 | 4.9 | 0.1 |
|  |  |  |  |  |  |  |
|  |  |  |  |  |  |  |
|  |  |  |  |  |  |  |
| **Model** | **LL** | **K** | **n/K** | **AICc** | **Δ_i_** | **w_ic_** |
| ***temperature + rainfall + temperature:rainfall*** | **-90.2** | **4** | **40.0** | **188.7** | **0.0** | **0.9** |
| *temperature + rainfall* | -94.6 | 3 | 53.3 | 195.1 | 6.4 | 0.1 |
| *temperature* | -96.1 | 2 | 80.0 | 196.3 | 7.6 | 0.0 |
| *1* | -96.9 | 1 | 160.0 | 195.8 | 7.1 | 0.0 |
|  |  |  |  |  |  |  |
| *year* | -93.6 | 11 | 15.1 | 211.0 | 7.5 | 0.0 |
| ***1*** | **-100.7** | **1** | **166.0** | **203.5** | **0.0** | **1.0** |
|  |  |  |  |  |  |  |
| *NAO* | **-98.4** | **2** | **83.0** | **200.9** | **0.0** | **0.8** |
| *1* | -100.7 | 1 | 166.0 | 203.5 | 2.6 | 0.2 |
|  |  |  |  |  |  |  |
| ***NAO + farms*** | **-86.4** | **3** | **50.3** | **179.0** | **0.0** | **0.9** |
| *NAO* | -90.1 | 2 | 75.5 | 183.9 | 4.9 | 0.1 |
| *1* | -92.0 | 1 | 151.0 | 186.0 | 7.0 | 0.0 |
|  |  |  |  |  |  |  |
| *NAO + farms + temperature + rainfall+temperature:rainfall* | -82.7 | 6 | 25.0 | 178.5 | 1.1 | 0.2 |
| *NAO + farms + temperature + rainfall* | -83.6 | 5 | 30.0 | 177.7 | 0.3 | 0.2 |
| *NAO + farms + temperature* | -84.6 | 4 | 37.5 | 177.6 | 0.2 | 0.3 |
| ***NAO + farms*** | **-85.6** | **3** | **50.0** | **177.4** | **0.0** | **0.3** |
| *NAO* | -89.1 | 2 | 75.0 | 182.2 | 4.8 | 0.0 |
| *1* | -90.8 | 1 | 150.0 | 183.6 | 6.2 | 0.0 |
|  |  |  |  |  |  |  |
|  |  |  |  |  |  |  |
| c) Final logistic equation |  |  |  |  |  |  |
|  |  |  |  |  |  |  |
| ***NAO + farms*** | **-86.4** | **3** | **50.3** | **179.0** | **0.0** | **0.9** |
| *NAO* | -90.1 | 2 | 75.5 | 183.9 | 4.9 | 0.1 |
| *1* | -92.0 | 1 | 151.0 | 186.0 | 7.0 | 0.0 |
|  |  |  |  |  |  |  |
|  |  |  |  |  |  |  |

LL, Maximized log-likelihood; K, Number of estimated parameters; n/K, number of observations/K; AICc, Akaike’s Information Criterion ; Δi, difference between AIC and the lowest AIC value; w_ic_, Akaike weight. Values in bold correspond to the selected models.
